# Cell death in sepsis: unveiling new perspectives on organ dysfunction

**DOI:** 10.3389/fcell.2026.1823003

**Published:** 2026-04-23

**Authors:** Runze Jiang, Pingping Cai

**Affiliations:** 1 Shandong University of Traditional Chinese Medicine, Jinan, Shandong, China; 2 Department of Traditional Chinese Medicine, Shandong Provincial Hospital Affiliated to Shandong First Medical University, Jinan, China

**Keywords:** immune dysregulation, organ dysfunction, programmed cell death, sepsis, therapeutic targets

## Abstract

Despite significant advances in modern intensive care, sepsis remains a life-threatening inflammatory disease worldwide. High mortality is mainly due to multiple organ dysfunction syndrome (MODS). Emerging research has shifted the focus to regulating cell death (RCD) pathways, particularly siderophosis and pyrophosphosis, which are considered major drivers of immune damage. In addition to inducing cell death, these RCD mechanisms exacerbate the inflammatory response, disrupt physiological balance, and directly accelerate MODS-related failure. Key pathogenic mechanisms include gasdermin D (GSDMD)-mediated scorching, cytokine storming, iron destruction-related lipid peroxidation impairment of cardiac function, and immune apoptosis leading to severe immunosuppression. While pharmacologically targeting these pathways has therapeutic potential, striking a balance between efficacy and infection defense remains challenging. Therefore, improving sepsis treatment requires a time-adaptive, multi-target approach. Additionally, identifying reliable biomarkers is crucial for accurate diagnosis, ultimately improving patient outcomes.

## 1 Introduction

Sepsis is a systemic inflammatory response syndrome triggered by infection. The core pathological mechanisms of sepsis include imbalance of the inflammatory cascade, abnormal immune regulation, and coagulation system dysfunction. The synergistic effect of the three can induce septic shock, severe sepsis, and MODS. Once the disease progresses to the MODS or septic shock stage, the patient’s mortality rate will increase sharply (Opal et al., 2015; O'Brien et al., 2007; Arora et al., 2023). Despite the continuous advancement of modern intensive care technology, sepsis remains an important cause of patient death worldwide (Kheterpal et al., 2022). The Global Burden of Disease Report shows that in 2017, there were 48.9 million confirmed cases of sepsis worldwide, and the disease fatality rate reached 22.5%. This number is equivalent to approximately 20% of the total global deaths (Seymour et al., 2019). The prognosis of sepsis depends on the dynamic balance of pro-inflammatory and anti-inflammatory responses (Hotchkiss et al., 2013). Existing treatment options mainly include antibiotic application and fluid resuscitation. Early empiric broad-spectrum antimicrobial therapy is crucial, but can also lead to antibiotic misuse and resistance (Niede et al., 2021). At present, the treatment of septic shock has not been individualized, and new biomarkers need to be systematically discovered to optimize early diagnosis, prognosis assessment and drug development (Pandey, 2024).

Given this high mortality, recent research has pivoted toward understanding how dysregulated cell death pathways contribute to organ failure. Cellular dysfunction is increasingly being recognized as an important factor in the development of organ failure (Lelubre and Vincent, 2018). When sepsis occurs, the response ability of immune cells is regulated by different apoptotic signals and changes dynamically. Apoptosis-mediated cell death directly promotes microcirculation disorders and MODS, and leads to secondary infection or immunocompromise due to loss of immune cells (Cao et al., 2019). Autophagy is a lysosome-mediated process of selective degradation of macromolecules and damaged organelles. It is essential for maintaining cellular homeostasis, and regulates the differentiation, renewal and energy metabolism of immune cells in sepsis (Kumar, 2020), and prevents MODS (Wang et al., 2019). Pyroptosis is regulated by the GSDM protein family, which can trigger cell lysis and release pro-inflammatory factors to play a pathogenic role (Guo et al., 2021). Necroptosis is a programmed cell death (PCD) triggered by multiple cytokines, death receptors, and pattern recognition receptors (Wallach et al., 2016). Ferroptosis is a form of cell death that is dependent on iron ions. Its core manifestations are disordered iron metabolism and accumulation of iron-dependent lipid peroxidation products (Xl et al., 2022).

**FIGURE 1:**
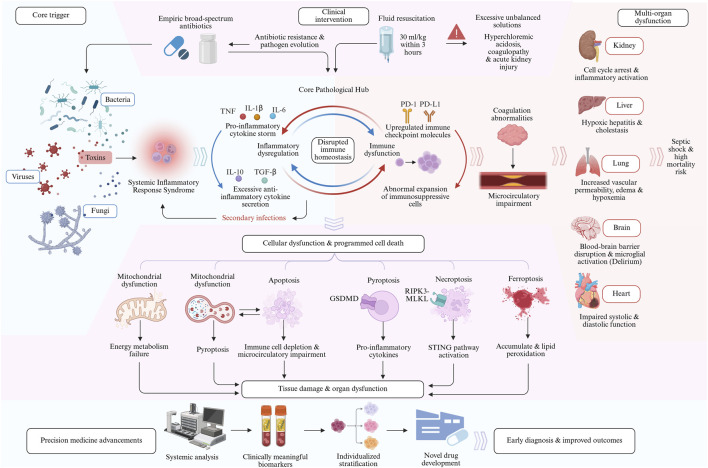
Graphical abstract. This figure systematically illustrates the comprehensive pathological progression of sepsis from initial infection to multiple organ dysfunction. Pathogen invasion triggers Systemic Inflammatory Response Syndrome, establishing a central pathological hub characterized by pro-inflammatory cytokine storms, immune dysregulation, and secondary infections. This hub subsequently drives programmed cell death and cellular dysfunction, ultimately resulting in tissue damage, multi-organ failure, and high mortality risk. The diagram identifies critical windows for early clinical interventions, including empiric antibiotics and fluid resuscitation, while forecasting future breakthroughs in precision medicine, clinically meaningful biomarkers, and individualized therapeutic strategies to improve early diagnosis and patient outcomes.

Sepsis often involves multiple organ systems, leading to a series of serious clinical complications. The kidney is one of the most common target organs of sepsis, and the occurrence of sepsis-associated acute kidney injury (SA-AKI) significantly increases the morbidity and mortality of patients, becoming a key factor affecting prognosis (Poston and Koyner, 2019). Sepsis-associated liver injury (SALI) is a serious complication of sepsis, mainly caused by microcirculation disorders, entero-liver axis and inflammatory response (Chen J. et al., 2024). Sepsis-associated acute lung injury (SA-ALI) and acute respiratory distress syndrome (ARDS) are severe pulmonary complications secondary to sepsis, characterized by refractory hypoxemia, dyspnea, and pulmonary edema (Ortiz et al., 2019; Lindstedt et al., 2024). Delirium is the main clinical manifestation of sepsis-associated encephalopathy (SAE), which leads to increased mortality and may leave long-term cognitive impairment or focal neurological dysfunction (Tauber et al., 2021). Sepsis-induced myocardial dysfunction (SIMD) is characterized by bilateral cardiac systolic and diastolic dysfunction, and its underlying mechanisms include inflammatory mediators, cardiomyocyte contractility dysfunction, mitochondrial dysfunction, reduced energy metabolism, and cell death (Guo et al., 2022; Lin et al., 2020) (Figure 1, summary of pathological mechanisms of sepsis).

This review aims to systematically elucidate the core role of cell death in sepsis organ damage, and reveal how different death modes such as apoptosis, autophagy, pyroptosis, necroptosis and ferroptosis participate in the pathological process of MODS by regulating inflammatory response, immune homeostasis and metabolic imbalance, and provide new ideas for clinical diagnosis and treatment. Although existing studies have elucidated some aspects of the pathophysiology of sepsis, there is still a lack of overall understanding of the organ-specific injury mechanisms mediated by cell death.

## 2 Cell death mechanisms in sepsis

### 2.1 Apoptosis

Apoptosis is a widely studied form of PCD in sepsis, mainly manifested by cell shrinkage, chromatin agglutination, and maintenance of cell membrane integrity (Choi H. et al., 2025; Vermeulen et al., 2005) In sepsis, excessive apoptosis of immune cells, especially lymphocytes and dendritic cells, is considered an important cause of immunoparalysis (Hotchkiss and Nicholson, 2006). Studies have also reported that in the early stages of sepsis, CD4 T lymphocytes and B lymphocytes have increased apoptosis, absolute peripheral blood lymphocyte counts have dropped sharply, and persistent lymphopenia is directly associated with increased mortality in sepsis patients (Pei et al., 2022).

The mitochondrial pathway and the death receptor pathway are the two main pathways of apoptosis (Gupta, 2001). The mitochondrial apoptosis pathway is controlled and regulated by the Bcl-2 protein family. Bax and Bak, as members of the Bcl-2 protein family, are activated and accumulate on the outer mitochondrial membrane stimulated by apoptosis, thereby promoting the release of cytochrome c, which in turn triggers the Caspase cascade (Xiao et al., 2023). The death receptor pathway is triggered by the Fas ligand and tumor necrosis factor (TNF)-related apoptosis-inducing ligand (TRAIL), which induces apoptosis through the activation of caspase-8 (Wang and Labzin, 2023; He et al., 2022).

**FIGURE 2:**
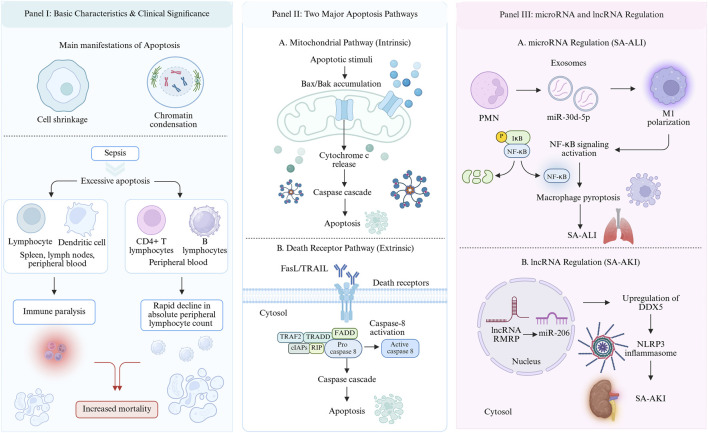
The mechanisms of apoptosis in sepsis. Sepsis stimulates the aggregation of pro-Bax/Bak on the outer mitochondrial membrane, releasing cytochrome c to trigger the caspase cascade. Alternatively, it activates the death receptor-caspase-8 pathway via FasL/TRAIL, leading to massive lymphocyte apoptosis, a sharp decrease in peripheral blood lymphocyte counts, and the induction of immune paralysis. Simultaneously, PMN exosomal miR-30d-5p promotes M1 polarization and macrophage pyroptosis, exacerbating SA-ALI. Additionally, lncRNA RMRP upregulates DDX5 via miR-206, activating the NLRP3 inflammasome and amplifying SA-AKI. The apoptosis regulatory network emerges as a critical determinant of prognosis.

In recent years, scientific research has increasingly focused on elucidating the complex regulatory mechanisms of apoptosis during sepsis, with particular emphasis on the role of long non-coding RNAs (lncRNAs) and microRNAs (miRNAs) (Ho et al., 2016). A notable example involves polymorphonuclear neutrophils (PMN)-derived exosomal miR-30d-5p, which exacerbates SA-ALI by promoting M1 macrophage differentiation and activating pyroptotic cell death via NF-κB pathway modulation (Jiao et al., 2021). Moreover, the lncRNA RMRP acts to exacerbate SA-AKI by modulating the miR-206/DDX5 axis, which subsequently triggers the activation of the NLRP3 inflammasome (Zhang et al., 2021) (Figure 2, molecular pathways governing apoptotic modulation in sepsis).

### 2.2 Autophagy

Autophagy is a key part of balancing energy needs when nutrients are scarce. It is also important for cell survival in many ways (Glick et al., 2010; He et al., 2020). While it usually works to keep cells alive, any change in the autophagic pathway or an increase in the flow can lead to death (Liu S. et al., 2023). While the hyperactivation observed under extreme stress may prove maladaptive, ultimately leading to autophagic cell death, basal autophagic activity plays a defensive role during sepsis by sequestering toxic oxidized proteins (Sang et al., 2021).

Mammalian target of rapamycin (mTOR) signaling usually works to stop autophagy, but when Beclin-1 is overexpressed in heart tissues, it can get around this block and keep autophagic flow going even when the body is under septic stress (Sun et al., 2018). At the same time, targeted degradation of mitochondria, known as mitophagy, has emerged as a key factor in sepsis-induced injury (Patoli et al., 2020). High levels of macrophage migration inhibitory factor (MIF) can directly bind to PINK1, disrupt its interaction with Parkin and impair the initiation of mitochondrial autophagy, thereby aggravating SA-AKI (Li et al., 2024). Autophagy dysfunction can lead to hyperinflammation and NLRP3 inflammasome overactivation (Biasizzo and Kopitar-Jerala, 2020), and in SA-ALI, the SIRT1 signaling pathway effectively inhibits the activation of NLRP3 and stimulator of interferon genes (STING) pathways by enhancing mitochondrial autophagy mediated by endosomes of lung endothelial cells, which provides a potential target for related therapies (Jiang et al., 2024).

However, the protective boundaries of autophagy are finite and context-dependent. When sepsis progresses to severe stages or involves persistent hypoxia and oxidative stress, excessive or dysregulated autophagy can transition into a destructive force. Hyperactivation of autophagy may lead to excessive self-digestion of essential organelles and cytoplasmic components, culminating in autophagic cell death, a form of regulated cell death distinct from apoptosis that contributes to tissue atrophy and organ dysfunction (Liu and Levine, 2015; Yu et al., 2006).

### 2.3 Necroptosis

Necroptosis is a form of PCD. Receptor-interacting protein kinase 3 (RIPK3) and mixed lineage kinase domain-like protein (MLKL) are critical mediators of necroptosis; activation of RIPK3 and MLKL leads to plasma membrane rupture and excessive inflammation. Studies have shown that the RIPK3-MLKL-mediated necroptotic signaling pathway can enhance the activation of the STING pathway, thereby exacerbating the lethality of sepsis (Zhang et al., 2023; You et al., 2025).

There is a profound association between activation of necrotizing apoptosis and the characteristics of worsening inflammatory states in sepsis. High mobility group protein B1 (HMGB1) is often recognized as a signature molecule of damage-associated molecular patterns (DAMPs), and its impact on the immune microenvironment upon entry into the extracellular environment, whether actively or passively released, is present (Chen et al., 2022). HMGB1 levels are associated with sepsis-related mortality and expression patterns of RIPK3 and MLKL, suggesting that this protein is involved in the regulatory mechanism of necrotizing apoptosis (Yoo et al., 2021).

In addition, the multiple functions of necrotizing apoptosis in different organs have attracted widespread attention. Necrotizing aptoposis of alveolar epithelial cells (AECs) is considered an important pathological event in SA-ALI (Hagiwara et al., 2020). In preclinical models, therapeutic strategies targeting necrotizing apoptosis, such as MLKL inhibitors (Necrosulfoamide), RIPK1 inhibitors (Necrostatin-1 or Nec-1), and RIPK3 inhibitors (GSK'840), have shown good efficacy, opening up new avenues for the treatment of SA-ALI (Cao et al., 2024; Song et al., 2023).

### 2.4 Ferroptosis

Specifically, ferroptosis is a newly discovered form of regulated necrosis characterized by excessive iron accumulation and extensive oxidative degradation of lipids (Li et al., 2020). Under physiological conditions, cellular homeostasis is maintained by the antagonism between an oxidative system comprising iron ions, the Fenton reaction, and reactive oxygen species (ROS) and an antioxidant defense system consisting of glutathione peroxidase 4 (GPX4), glutathione, System Xc^−^, and several newly discovered pathways (Dixon et al., 2012).

Central to the systemic aggravation of sepsis is the process of ferroptosis, a notion supported by its capacity to modulate iron dynamics. Ferroptosis releases iron from inside cells, which helps bacteria grow because they need iron to do so. This creates an environment that is good for infection. The excess iron works as a pro-oxidant catalyst for lipid peroxidation and the production of ROS. This creates a never-ending cycle that speeds up the spread of localized infections to septicemia and systemic MODS (Chen et al., 2021).

Moreover, the involvement of ferroptosis in SIMD has garnered significant attention; for instance, modulating the expression of the anti-lipid peroxidative protein GPX4 has been shown to improve cardiac contractile function following ischemia/reperfusion injury (Zou et al., 2024; Zhang et al., 2024a). Treatments for ferroptosis, such as lipid antioxidants (ferrostatin-1), iron chelators (deferoxamine), and GPX4 activators, show promise for reducing organ damage and providing promising opportunities for the clinical treatment of sepsis (Akalay and Hosgood, 2023).

### 2.5 Pyroptosis

Pyroptosis is a major pro-inflammatory PCD that leads to sepsis, with the gasdermin protein family and inflammasomes serving as its primary regulators. Among these, GSDMD functions as the pivotal hub protein connecting both canonical and non-canonical inflammasome pathways (Vasudevan et al., 2023). This kind of cell death is marked by the cell’s membrane breaking and the cell growing quickly, both of which cause inflammatory substances to be released from inside the cell (Rao et al., 2022). Under septic conditions, the NLRP3 inflammasome, a multiprotein complex, acts as a crucial molecular sensor capable of detecting various pathogen-associated molecular patterns (PAMPs) and DAMPs, consequently triggering pyroptosis (Li N. et al., 2019; Li et al., 2025).

Moreover, the regulatory mechanisms of pyroptosis have also attracted extensive attention. Pyroptosis closely associated with inflammatory responses involves inflammasome formation, activation of caspase-1, and cleavage of the N-terminal fragment of GSDMD, leading to plasma membrane rupture and cell death, which may be implicated in the pathogenesis of SA-AKI (Li et al., 2022). Notably, pyroptosis promotes the extracellular release of IL-1β/IL-18, initiating inflammatory responses, and facilitates the release of intracellular DAMPs, further inducing pyroptosis in neighboring cells, thereby establishing a positive feedback loop (Ding et al., 2021).

**FIGURE 3:**
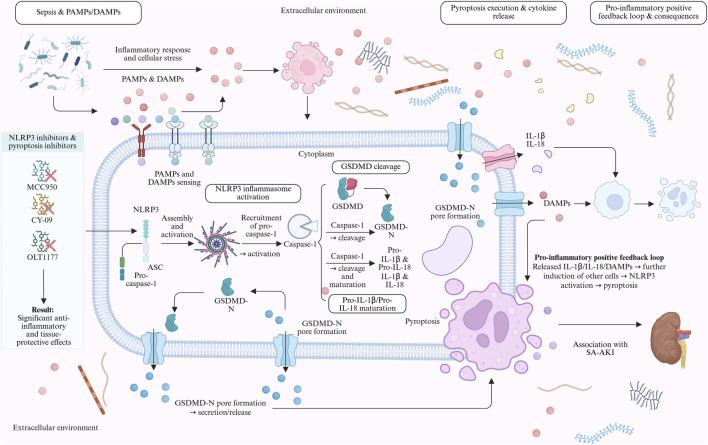
The mechanism of pyroptosis in sepsis. PAMPs/DAMPs activate the NLRP3 inflammasome, recruiting pro-caspase-1 and cleaving it into active caspase-1. The latter then cleaves GSDMD to release GSDMD-N, which forms pores on the cell membrane, leading to cell swelling, rupture, and the release of IL-1β/IL-18 and DAMPs. These released factors further stimulate neighboring cells' NLRP3, creating a pyroptosis-inflammation positive feedback loop that amplifies tissue damage. NLRP3 inhibitors such as MCC950 and pyroptosis inhibitors can block this loop, alleviating complications like SA-AKI, but a balance must be maintained between anti-infection effects and immune suppression.

Various NLRP3 inhibitors (MCC950, CY-09, OLT1177) and pyroptosis inhibitors have demonstrated significant anti-inflammatory and tissue-protective effects in preclinical models (Wang et al., 2025a), although a balance between anti-inflammatory and anti-infective requirements remains necessary (Figure 3, mechanism of pyroptosis in sepsis).

### 2.6 Other forms of cell death

In addition to the major forms of cell death mentioned above, other types of cell death are also involved in sepsis, such as NETosis and efferocytosis. Neutrophil extracellular traps (NETs) can capture and kill pathogens, including bacteria, viruses, fungi, and protozoa; NETosis represents a specialized form of cell death responsible for NET formation (Huang J. et al., 2022). Research indicates that NETs facilitate bacterial clearance but also contribute to thrombosis and organ injury during sepsis (Alsabani et al., 2022). The human body eliminates billions of apoptotic cells daily through efferocytosis, a process critical for maintaining homeostasis. To sustain efferocytosis, phagocytes undergo rapid phenotypic changes enabling them to engulf and degrade apoptotic debris. Efferocytosis exhibits active anti-inflammatory properties and promotes the resolution of inflammation (Mehrotra and Ravichandran, 2022).

Recent advances have introduced the concept of PANoptosis, an integrated form of cell death encompassing pyroptosis, apoptosis, and necroptosis, providing insight into the overlapping cellular mortality observed during sepsis progression. Caspase-8 serves as a key regulator of this process, with its activation or deficiency determining cell survival or death fate in inflammatory conditions (Zhang et al., 2024b). The proposal of the PANoptosis concept reveals a higher-level integrated mechanism. ZBP1, functioning as a pattern recognition receptor, can simultaneously sense viral or fungal infections, interact with RIPK3 via its RHIM domain to promote necroptosis, and activate the NLRP3 inflammasome to drive pyroptosis (Gong T. et al., 2024). In a model of sepsis-associated encephalopathy, TLR9 activates PANoptosis through the p38 MAPK signaling pathway; inhibition of p38 MAPK blocks apoptosis and pyroptosis but paradoxically triggers necroptosis. When necroptosis is suppressed, apoptosis and pyroptosis are subsequently reactivated, forming a complex compensatory network (Zhou et al., 2022). This “trade-off” compensatory relationship suggests that inhibition of a single pathway may lead to a shift in cell death mode rather than promoting cell survival.

Moreover, the interconversion among different modes of cell death and their significance in organ injury during sepsis represent current research frontiers (Table 1, major types of cell death in sepsis and their characteristics). Notably, these cell death pathways interact through complex signaling networks that exacerbate inflammatory responses and lead to collapse of the organ microenvironment, thereby driving the development and progression of MODS (Liu and Liang, 2025). For instance, deficiency or inhibition of caspase-8 can redirect macrophages from apoptosis toward necroptosis (Pilot et al., 2025), while GSDMD mediates crosstalk between pyroptosis and necroptosis, potentially promoting apoptosis via enhanced endoplasmic reticulum (ER) stress or accelerating necroptosis by forming pores on mitochondrial membranes (Cheng et al., 2024).

**TABLE 1:** Major types of cell death in sepsis and their characteristics.

Type of cell death	Key regulators	Morphological features	Immunomodulatory effects	Refrences
Apoptosis	Caspase-3/8/9, Bcl-2 family (Bax/Bak)	Cell shrinkage, chromatin condensation, apoptotic bodies	Immunologically silent (prevents inflammation), but lymphocyte apoptosis leads to immune paralysis	Hotchkiss and Nicholson (2006), Xiao et al. (2023)
Autophagy	mTOR, Beclin-1, PINK1/Parkin	Autophagosome formation, lysosomal degradation	Double-edged sword: clears damaged organelles vs. excessive autophagy triggers cell death	He et al. (2020), Li et al. (2024)
Necroptosis	RIPK3, MLKL	Plasma membrane rupture, cellular content release	Highly proinflammatory; DAMPs exacerbate organ injury	You et al. (2025), Yoo et al. (2021)
Ferroptosis	GPX4, SLC7A11, ACSL4	Mitochondrial cristae reduction, lipid ROS accumulation	Iron dysregulation drives oxidative damage, worsens infections	Xl et al. (2022), Zou et al. (2024)
Pyroptosis	NLRP3, GSDMD, Caspase-1/4/11	Cell swelling, membrane pore formation, inflammatory cytokine release	Directly fuels cytokine storms (IL-1β/IL-18)	Vasudevan et al. (2023), Li et al. (2025)

The pathogenesis of sepsis is closely associated with aberrant activation of multiple cell death pathways, including apoptosis, autophagy, pyroptosis, necroptosis, and ferroptosis. Their interactions not only disrupt immune homeostasis but also intensify organ damage and inflammatory responses, collectively fueling disease progression. Targeting these interconnected pathways may offer novel personalized therapeutic strategies for sepsis.

## 3 Interaction between cell death and immune responses

### 3.1 Regulation of inflammatory responses by cell death

In the early stages of sepsis, the dominant pathogen overactivates the body’s innate immune system, producing an excessive inflammatory response (Huang Z. et al., 2022), and multiple forms of cell death are involved in the regulation of the inflammatory response through their respective pathways. In the late stage of sepsis, the immunosuppressive effects of pyroptosis become increasingly evident. Sustained pyroptosis leads to massive exhaustion of immune cells, resulting in immunoparalysis. Pyroptosis in monocytes, induced via caspase-1 or caspase-11-dependent pathways, causes a state of “endotoxin tolerance,” characterized by diminished secretion of pro-inflammatory cytokines (TNF-α, IL-6, IL-12) and elevated levels of anti-inflammatory mediators (IL-10, IL-1 receptor antagonist), thereby increasing the risk of nosocomial infections and mortality (Islam et al., 2024). Concurrently, pyroptosis and apoptosis of T lymphocytes jointly contribute to lymphopenia, particularly the depletion of CD4^+^ T cells and B cells, impairing adaptive immune responses and defense against secondary infections (Lv et al., 2022).

Pyroptosis and apoptosis are both programmed deaths, but pyroptosis causes cell membrane rupture and rapidly releases a large amount of inflammatory content into the extracellular space, thereby inducing inflammation, while apoptosis is immunosile, and the contents of dead cells are encased in apoptotic bodies (Ketelut-Carneiro and Fitzgerald, 2022). Apoptosis of monocytes and macrophages promotes immunosuppression in sepsis, manifested by reduced pro-inflammatory capacity, increased anti-inflammatory mediators, and dysregulation of HLA-DR expression leading to increased susceptibility and mortality, leading to a poor prognosis (Islam et al., 2024). In sepsis-associated ARDS, due to defective or overloaded burial capacity of alveolar macrophages, apoptotic neutrophils may accumulate, leading to secondary necrosis and release of inflammatory mediators into the alveolar cavity, which may lead to a persistent inflammatory response (McCubbrey and Curtis, 2013; Mahida et al., 2021).

In contrast, pyroptosis is a potent form of cell death that actively triggers inflammation. This process depends on pore formation by proteins of the GSDM family. In sepsis, lipopolysaccharide (LPS) activates caspase-4/5/11, leading to cleavage of GSDMD and subsequent induction of pyroptosis. Moreover, GSDMD cleavage also causes potassium efflux, which promotes assembly of the NLRP3 inflammasome and cleavage of pro-IL-1β and pro-IL-18 (Liu et al., 2024) thereby driving a cytokine storm. Recent studies have revealed that under certain conditions apoptotic cells may undergo a transition toward a pyroptosis-like cell death process termed “efferocytosis-associated pyroptosis”, which can further amplify inflammatory responses. This phenomenon may represent a novel mechanism underlying uncontrolled inflammation in sepsis (Muendlein et al., 2025).

### 3.2 Alterations of immune in sepsis

The immune status of sepsis is characterized by a distinct biphasic character, with a high inflammatory response in the early stages and immunosuppression in the late stages (van der Poll et al., 2017). The early stage of hyperinflammation is associated with a pro-inflammatory response aimed at killing invading pathogens (Hotchkiss and Karl, 2003). Immunosuppression is a typical pathophysiological feature of advanced sepsis, which is mainly manifested by weakened cell proliferation, increased lymphocyte apoptosis, and excessive release of anti-inflammatory cytokines *in vivo* (Hotchkiss et al., 2013; Long et al., 2020). This immune dysfunction is closely related to the death and functional alterations of several immune cells.

Neutrophils are one of the first immune cells to be activated in sepsis, and their apoptosis, pyroptosis, or NETosis processes significantly affect the course of sepsis and are associated with MODS (Cheng et al., 2020). Significant functional abnormalities, such as decreased oxidative burst, impaired migration, and attenuated chemotaxis, are present in neutrophils isolated from septic patients and collectively impair the ability to eliminate pathogens (Demaret et al., 2015; Hampson et al., 2017). Furthermore, the aberrant accumulation of NETs damages endothelial cells, activates platelets, and delivers tissue factor. These processes promote thrombosis and coagulopathy, thereby exacerbating organ dysfunction in sepsis (Iba and Levy, 2018).

Patients with sepsis-associated lymphopenia face an increased risk of secondary complications, leading to prolonged healthcare duration and elevated mortality rates (Finfer et al., 2023; Liu B. et al., 2022). Owing to their pivotal role in adaptive defense, T lymphocytes are active throughout the immune cascade. Interestingly, one of the hallmarks of immunosuppression associated with sepsis is the development of T cell exhaustion. Given this, measures to protect T cell populations and their functionality could provide a promising means of reversing immune paralysis (Venet and Monneret, 2018). In particular, T cells in septic mice exhibit higher levels of pro-apoptotic Bim and Puma, which can be suppressed to greatly increase T cell persistence and host resilience (Brahmamdam et al., 2009).

**FIGURE 4:**
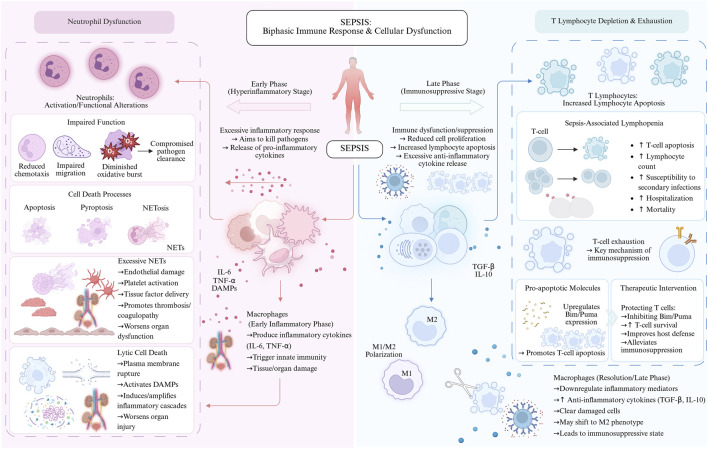
The immune status in sepsis exhibits a biphasic pattern characterized by early hyperinflammation followed by a late immunosuppressive phase. Sepsis is characterized by a biphasic immune imbalance: in the early phase, delayed neutrophil apoptosis and excessive NET release exacerbate endothelial injury and hypercoagulability, while in the late phase, T cell exhaustion and apoptosis, along with M2 polarization and lytic death of monocytes/macrophages, lead to the release of DAMPs, creating a vicious cycle of concurrent immunosuppression and persistent inflammation that drives multiorgan failure.

Significant heterogeneity exists within the monocyte and macrophage populations during septic progression. Initially, the production of TNF-α and IL-6 by these cells orchestrates an innate immune response aimed at neutralizing pathogens. Nevertheless, the inflammatory surge orchestrated by macrophages is frequently associated with detrimental effects on organ function. Subsequent to this, a shift toward a pro-resolving phenotype occurs, characterized by a decrease in inflammatory output and an increase in IL-10 and TGF-β levels (Daley et al., 2010). Systemic immune paralysis in the chronic stages of sepsis is further exacerbated by macrophage polarization toward an M2 state (Tao et al., 2024). Significantly contributing to the pathophysiology of septic organ injury is the induction of lytic cell death and the resulting rupture of the plasma membrane, which can set off an enhanced inflammatory cascade via ER-dependent pathways (Zhong et al., 2023) (Figure 4, the biphasic immune response in sepsis).

Different pathogens precisely regulate host cell death pathways through their unique PAMPs and virulence factors, thereby shaping the pathological progression of sepsis. Gram-negative bacteria such as *Pseudomonas aeruginosa* and *Yersinia pestis* primarily activate the caspase-4/5/11 non-canonical inflammasome pathway via LPS, inducing GSDMD-mediated pyroptosis. Meanwhile, their type III secretion system (T3SS) effector proteins (YopJ) can trigger RIPK1-dependent PANoptosis by inhibiting the TAK1 kinase, a concerted form of cell death that simultaneously activates pyroptosis, apoptosis, and necroptosis (Xiao J. et al., 2025).

Fungal pathogens such as *Candida* albicans and Aspergillus fumigatus induce cell death through distinct mechanisms: they recognize fungal nucleic acids via the Zα2 domain of ZBP1, thereby activating a PANoptosome complex composed of NLRP3, RIPK3, and caspase-8, which simultaneously triggers pyroptosis, apoptosis, and necroptosis. This tripartite mode of cell death is particularly critical in invasive fungal infections in immunocompromised hosts (Banoth et al., 2020). In contrast, viruses such as influenza virus, SARS-CoV-2, and HSV-1 primarily induce expression of interferon-stimulated genes through nucleic acid sensors (RIG-I, MDA5, cGAS-STING), while also activating necroptosis via the ZBP1-RIPK3-MLKL axis. When apoptosis is suppressed by viruses, necroptosis serves as a backup defense mechanism; however, excessive ZBP1 activation can lead to ARDS and SA-AKI. Furthermore, certain viruses such as coxsackievirus B3 can induce necroptotic death in cardiomyocytes by inhibiting caspase-8 activity, resulting in viral myocarditis (Xu et al., 2024).

### 3.3 Cell death signaling pathways and immune evasion

The immunosuppressive state in advanced sepsis is often driven by mechanisms such as T cell depletion, which is characterized by upregulation of immune checkpoint molecules, such as PCD protein 1 (PD-1) and its ligand PD-L1, and the PD-1/PD-L1 axis inhibits T cell receptor signaling, reduces T cell proliferation and cytokine production, and ultimately leads to immune paralysis, making patients more susceptible to secondary infections (Garduno and Martín-Loeches, 2024).

During apoptosis, caspases have traditionally been divided into apoptosis-associated and inflammation-related types, but their functions have crossed over (Zhang et al., 2025). Inflammation-associated caspases (caspase-1) mediate pyroptosis, while apoptosis-associated caspases (caspase-3, caspase-7, and caspase-9) not only regulate immune signaling, but also reduce the production of type I interferons (IFN-I) by inhibiting the cGAS-STING pathway activated by mitochondrial DNA (mtDNA), thereby maintaining the immune silencing state of cells (Zheng et al., 2023), indicating that they have both the dual functions of death execution and immune regulation.

Furthermore, impaired autophagic flux, characterized by the accumulation of autophagosomes due to lysosomal dysfunction or fusion defects, creates a pro-inflammatory environment through the sustained activation of the NLRP3 inflammasome and release of damage-associated molecular patterns (Saitoh et al., 2008; Nakahira et al., 2011). This paradoxical shift is observed in the later stages of sepsis, where autophagy insufficiency in immune cells correlates with immunoparalysis and increased susceptibility to secondary infections (Patoli et al., 2020; Chang et al., 2015).

## 4 Sepsis-associated organ injury and cell death

### 4.1 Sepsis-associated acute kidney injury

The occurrence of SA-AKI involves mechanisms such as activation of inflammatory responses, vascular dysfunction, and cell cycle arrest and apoptosis, while current prevention and control measures are still based on non-specific interventions such as controlling infection and avoiding aggravation of kidney injury (Chang et al., 2022). In SA-AKI, the special metabolic properties of proximal tubular epithelial cells (TECs) make them abnormally sensitive to ischemia, hypoxia, and oxidative stress. These cells undergo significant metabolic reprogramming in sepsis, manifested by inhibition of mitochondrial fatty acid oxidation and oxidative phosphorylation in favor of glycolysis for energy supply. Although this metabolic transition is an early protective adaptation that provides ATP rapidly in response to the energy crisis, long-term glycolytic dependence can lead to tubular atrophy, interstitial fibrosis, and increased risk of progression to chronic kidney disease. Restoring oxidative metabolism by activating metabolic regulators such as PGC-1α, AMPK, and SIRT1 has become a potential therapeutic strategy for reversing SA-AKI (Wang T. et al., 2024). Deficiency of RP105 leads to worsening of SA-AKI by exacerbating macrophage-mediated inflammatory responses, oxidative stress, and ferroptosis, while maintaining RP105 expression can reduce ferroptosis and inflammation by regulating the HO-1/SLC7A11/GPX4 axis, thereby improving renal function and is expected to be a new therapeutic target for SA-AKI (Duo et al., 2025).

Proximal TECs exhibit marked susceptibility to ferroptosis due to their high mitochondrial density and metabolic reliance on fatty acid oxidation. Studies have shown that under septic conditions, proximal TECs undergo metabolic reprogramming from oxidative phosphorylation toward glycolysis, resulting in diminished capacity to clear lipid peroxides. Concurrently, ACSL4 and LPCAT3 are highly expressed in this cell type, promoting the incorporation of PUFA into membrane phospholipids and significantly increasing susceptibility to lipid peroxidation (Zou and Schreiber, 2020).

At the same time, activation of the caspase-11/GSDMD pathway controls the release of NETs from neutrophils during sepsis, and GSDMD inhibits the prevention of MODs during sepsis by blocking the formation of NETs. Notably, GSDMD, as a member of the GSDM family, has been shown to be a co-executive protein for a variety of regulatory cell deaths, including pyroptosis, NETosis, and necrotizing apoptosis, and its mediated cell membrane pore formation is a central link in sepsis-related organ damage. Targeting GSDMD can not only directly block pyroptosis of renal TECs, but also inhibit the release of NETs, thereby protecting kidney function in multiple dimensions (Ma et al., 2024).

Sepsis-induced NETosis not only captures pathogens by releasing an extracellular trapping network composed of DNA, histones, and granulin proteins, but also activates endothelial and epithelial cells as DAMPs, exacerbating the inflammatory cascade. NETs components can stimulate AIM2 inflammasome activation, further promote GSDMD cleavage and pyroptosis, forming a vicious cycle. The latest research confirms that there is a complex bidirectional regulation of NETosis and pyroptosis, and the GSDMD-N-terminal fragment can directly promote the formation of NETs, which in turn can enhance pyroptosis, which is the key pathological mechanism of sepsis MODS (Kashif et al., 2025). Inhibition of GSDMD can directly block pyroptosis and protect the kidneys from damage during sepsis (Silva et al., 2021).

Metabolism-immune interaction regulation also provides new ideas for SA-AKI intervention. L-arginine supplementation can produce NO through inducible nitric oxide synthase (iNOS), directly S-nitrosylation of NLRP3 protein, inhibit its oligomerization and ASC spot formation, reduce IL-1β and IL-18 levels in renal tissue by 45%–70%, and improve glomerular filtration rate (Tanuseputero et al., 2020). Notably, the iNOS-specific inhibitor L-NIL can completely block the protective effect of arginine, confirming that NO-mediated post-translational modification of NLRP3 is a key molecular mechanism. This nutritional immunomodulatory strategy is particularly clinically translatable in the sepsis state of metabolic reprogramming disorder, as it can precisely regulate the intensity of inflammation without comprehensively suppressing immune function.

### 4.2 Sepsis-associated liver injury

SALI can present as hypoxic hepatitis due to ischemic shock, cholestasis due to abnormal bile metabolism, or bile duct sclerosis (Xu et al., 2025). Hepatocyte-released DAMPs (HMGB1) synergistically interact with LPS to Kupffer cell lysosomes via the receptor for advanced glycation end-products (RAGE) pathway, mediating caspase-11-independent pyroptosis in sepsis (Hu et al., 2024). This non-canonical NLRP3 activation pathway is particularly significant in hepatic sinusoidal endothelial cells. Through paracrine signaling, the subsequent release of IL-1β triggers the TLR4/NF-κB signaling axis in nearby hepatic stellate cells (HSCs), transcriptionally upregulating NLRP3 expression and creating a positive feedback loop (Guo S. et al., 2024). The liver, receiving LPS and microbial products directly from the gut via the portal venous system, experiences continuous exposure of sinusoidal endothelial cells and Kupffer cells to high levels of endotoxins, thereby establishing a unique “gut-liver axis” microenvironment. This explains why NLRP3-mediated pyroptosis predominates in septic liver injury (Choi W. et al., 2025).

The regulatory network of ferroptosis in SALI is characterized by its multi-layered nature. As a core transcription factor, the impairment of nuclear factor erythroid 2-related factor 2 (Nrf2) nuclear translocation leads directly to the inhibition of GPX4 and SLC7A11 expression. Hepatocytes' nuclear Nrf2 protein levels sharply decline in cecal ligation and puncture (CLP) sepsis models, while lipid ROS build up and the ferroptosis marker PTGS2 is markedly upregulated. On the other hand, this process can be reversed by the Nrf2 activator tBHQ, which lowers the amount of iron in the liver and significantly increases survival rates (Zhong et al., 2024). Additionally, Maresin 1 protects the liver by suppressing the MAPK/NF-κB pathway to lessen NLRP3-mediated pyroptosis and activating the Nrf2/SLC7A11/GPX4 pathway to prevent ferroptosis. In LPS/D-GalN models, Maresin 1 administration results in reduced hepatic necrotic areas and diminished neutrophil infiltration (Guo Y. et al., 2024). Notably, aldehyde dehydrogenase 2 (ALDH2) alleviates hepatocyte ferroptosis and the elevation of liver enzymes by clearing the lipid peroxidation product 4-HNE, thereby preventing the inactivation of the core Nrf2/SLC7A11/GPX4 regulatory axis (Li L. et al., 2023). Pretreatment with the ALDH2 agonist Alda-1 restores GPX4 expression to 85% of normal levels and reduces serum ALT and AST levels by more than 60%.

Recent studies have elucidated the redox regulatory role of the TXNIP/TRX-1/GPX4 axis. Under septic stress, TXNIP dissociates from thioredoxin-1 and binds directly to GPX4, facilitating its proteasomal degradation. Curcumin maintains GPX4 stability by blocking the TXNIP-GPX4 interaction, thereby inhibiting hepatocyte ferroptosis (Chen K. et al., 2024).

### 4.3 Sepsis-associated acute lung injury

SA-ALI and ARDS are characterized by the disruption of the alveolar-capillary barrier (Ruan et al., 2025). Recent studies have demonstrated that pyroptosis of pulmonary vascular endothelial cells is a critical factor leading to increased vascular permeability (Kerr et al., 2019). There is a positive feedback loop between neutrophil NETosis and AEC death, according to research. In particular, histones in NETs cause direct harm to AECs, and DAMPs released from dying epithelial cells further activate neutrophils through the cGAS-STING pathway, which encourages more NETosis cycles. Therefore, a good treatment approach for SA-ALI is to target the NETs/cGAS-STING/necroptosis pathway in AECs (Sha et al., 2024). Moreover, the dual roles of autophagy are also organ-specific; while cardiac autophagy generally confers protection against sepsis-induced myocardial dysfunction, excessive autophagy in pulmonary endothelial cells may exacerbate acute lung injury by promoting barrier disruption and vascular leakage (Hsieh et al., 2011).

A key component in combining different forms of PCD is the cGAS-STING signaling axis. When AECs undergo pyroptosis or necroptosis and release mtDNA into the cytosol, cGAS interprets this as a warning sign and triggers STING. The latter creates a positive feedback loop known as “mtDNA-cGAS-STING-NLRP3” by directly upregulating NLRP3 expression in a PARP-1-dependent manner (Ning et al., 2020). Significantly, this harmful cycle is made worse in AECs by autophagy deficiency. A deficiency in Atg5 causes caspase-11 to become overactive and encourages the aberrant release of mtDNA, which in turn triggers the cGAS-STING-NLRP3 axis in macrophages and causes unchecked systemic inflammation (Wang et al., 2025b). Moreover, metabolic reprogramming modifies this axis’s sensitivity. Fumarate hydratase (FH) activity decreases mtDNA leakage and damages mitochondrial function. By maintaining the integrity of the mitochondrial membrane, FH overexpression reduces ALI, whereas FH knockdown dramatically increases the activity of the cGAS-STING pathway (Jiang et al., 2025).

Several important nodes for therapeutic intervention have been identified by recent research regarding the regulatory mechanisms of NETosis. It is known that the actin-related protein 2/3 (Arp2/3) complex controls neutrophil extracellular trap expulsion. Inhibiting Arp2/3 function lessens lung damage and NET release in models of abdominal sepsis (Ding et al., 2022). Moreover, the integrin Mac-1 (macrophage-1 antigen) regulates NETosis in two ways. In addition to worsening histone-induced SA-ALI, its overactivation promotes GSDMD-mediated pyroptosis, which increases NET formation (Mizuno et al., 2024). Interestingly, NETosis is intimately associated with mitochondrial functional status. Melatonin has been demonstrated to successfully lessen the severity of SA-ALI by blocking the necroptosis pathway and reducing the release of circulating mtDNA (Peng et al., 2025). This discovery relates the control of NETosis to mitochondrial homeostasis, indicating that preserving mitochondrial integrity could be a novel tactic for NETosis indirect inhibition.

One of the main areas of focus for SA-ALI intervention is PCD regulation. The main pathological mechanism of SA-ALI, according to reviews, is pulmonary hypoperfusion brought on by shock and hypotension brought on by sepsis, which in turn causes ischemic necrosis of alveolar endothelial cells.In contrast to inhibitors that target PCD, current treatments, including conventional measures like fluid resuscitation and antibiotics, offer limited efficacy (Deng et al., 2025). For example, by inhibiting both the formation of NETs and the activity of the NLRP3 inflammasome, the multi-herbal formula SH003 dramatically lowers mortality in septic mice (Kim et al., 2024). Apoptotic vesicles also create a new paradigm for cell-free therapy by suppressing platelet activation and NETosis via a mechanism mediated by CD73 (Tan et al., 2025).

### 4.4 Sepsis-associated encephalopathy

SAE frequently occurs as a complication in patients with severe sepsis. The underlying mechanisms of SAE involve several processes, including endothelial and microglial activation-induced inflammatory responses, breakdown of the blood-brain barrier, hypoxia, disturbances in neurotransmitter balance, activation of glial cells, and neuronal as well as axonal damage (Tauber et al., 2021). Notably, ferroptosis triggered by SAE has been detected in the hippocampus, where it manifests as increased iron accumulation, elevated ROS and MDA levels, decreased GSH concentration, enhanced ACSL4 expression, and reduced SLC7A11/GPX4 activity (Wang et al., 2022). Moreover, cylindromatosis (CYLD), a lysine 63-specific deubiquitinase, plays a critical role in this process; its downregulation exacerbates LPS-induced pyroptosis in astrocytes of mice, thereby promoting the development of postoperative cognitive dysfunction (Li L. et al., 2019). Additionally, the C-X-C motif chemokine receptor 5 (CXCR5) expressed by microglia in the hippocampus inhibits autophagy via the p38MAPK/NF-κB/STAT3 signaling pathway, which contributes to the occurrence of cognitive dysfunction (Shen et al., 2021).

Recent studies have highlighted the key role of lipocalin-2 (LCN2) in the regulation of neuroinflammation, which is significantly involved in the pathological process of SAE. Notably, microglia in the central nervous system, as resident immune cells, exhibit a significantly lower activation threshold for the NLRP3 inflammasome compared to peripheral macrophages, which is closely associated with the rapid progression of neuroinflammation in SAE. PIM-1 kinase specifically drives NLRP3 activation in microglia by promoting mitochondrial ROS production. In contrast, neurons themselves are relatively resistant to pyroptosis due to the absence of a complete inflammasome complex but may suffer indirect damage via paracrine pathways (Ji et al., 2025). In a mouse model of LPS-induced sepsis, the pathogenesis of SAE is closely related to neuronal degeneration, synaptic dysfunction, and cognitive decline, and is further exacerbated by mitochondrial dysfunction. Bioinformatics analysis and experimental data showed that LCN2 expression was significantly upregulated, which played a central regulatory role in immune response and neuroinflammatory pathway. Exogenous recombinant LCN2 can reproduce LPS-induced synaptic damage and cognitive impairment, while inhibition of LCN2 can reduce neuronal damage, mitochondrial damage, and cognitive deficits. These findings suggest that upregulation of LCN2 plays a key role in the progression of SAEs, targeting LCN2 inhibition or a potential strategy for treating neurocognitive impairment caused by sepsis (Guo et al., 2025). In addition, berberine may reduce neuroinflammatory responses and neuronal apoptosis by partially blocking the NF-κB/LCN2 signaling axis in the hippocampus of CLP model mice, thereby improving SAE (Gong H. et al., 2024).

The pathogenesis of SAE is closely related to mitochondrial dysfunction and elevated levels of oxidative stress. It is worth noting that the Src family tyrosine kinase Fgr kinase was significantly upregulated in the hippocampus of sepsis mice, and exacerbated mitochondrial damage, oxidative stress and neuroinflammatory response by regulating the SIRT1/PGC-1α signaling pathway. Treatment with Fgr inhibitors significantly improved the survival rate and cognitive and emotional dysfunction in mice with CLP-induced sepsis, while activation of the SIRT1/PGC-1α pathway further enhanced these protective effects (Liu Y. et al., 2023). Additionally, the deubiquitinating enzyme OTUD1 promotes the release of hexokinase 2 (HK2) from mitochondria, thereby driving pyroptosis in microglia and exacerbating the pathological progression of SAE (Jing et al., 2025). This finding underscores the critical interplay between metabolic reprogramming and cell death mechanisms in SAE. In particular, microglia-derived CXCL2 directly induces ferroptosis in hippocampal neurons via the CXCR2/Jun signaling axis, establishing a detrimental positive feedback loop mediated by microglia-neuron interactions (Yang et al., 2025). Natural compounds such as quercetin exert neuroprotective effects by blocking the CXCL2/CXCR2 pathway and inhibiting intercellular communication (Yang et al., 2024).

Recent studies have also revealed a deleterious cyclical relationship between SAEs and peripheral immunosuppression. Brain damage caused by sepsis is not only a dysfunction of a single organ, but also an important cause of immune regulatory network disorders. As the command center for the cholinergic anti-inflammatory pathway, the hypothalamic-pituitary-adrenal axis, and the sympathetic nervous system, the brain undergoes functional damage that leads to reduced numbers and abnormal responses of peripheral immune cells. This, in turn, intensifies uncontrolled neuroinflammation (Xiao D. et al., 2025). The bidirectional interaction between SAE and peripheral immune dysregulation suggests that disrupting this deleterious cycle may be an effective strategy for treating the immunosuppressive state induced by sepsis.

### 4.5 Sepsis-induced myocardial dysfunction

The mechanisms driving SIMD include excessive inflammatory responses, alterations in systemic and microvascular circulation, impaired nitric oxide synthesis, endothelial dysfunction, calcium dysregulation, abnormal cardiac autophagy, autonomic nervous system imbalance, metabolic reprogramming, and mitochondrial dysfunction (Lin et al., 2020). Ferroptosis is considered more likely to induce myocardial injury compared to other forms of cell death, primarily due to the high compatibility between its unique iron-dependent and lipid peroxidation mechanisms and the metabolic characteristics of the heart. Unlike other forms of cell death, ferroptosis generates hydroxyl radicals via the Fenton reaction, which directly attack phospholipids containing PUFA, resulting in oxidative damage to membrane systems, this process is particularly prominent in myocardial ischemia-reperfusion injury (Deng et al., 2024). Notably, cardiomyocytes are rich in mitochondria, which serve as key targets in ferroptosis. Inhibition of mitochondrial GPX4 leads to accumulation of mitochondrial lipid peroxidation, directly impairing the contractile function of cardiomyocytes (Chen et al., 2025).

**FIGURE 5:**
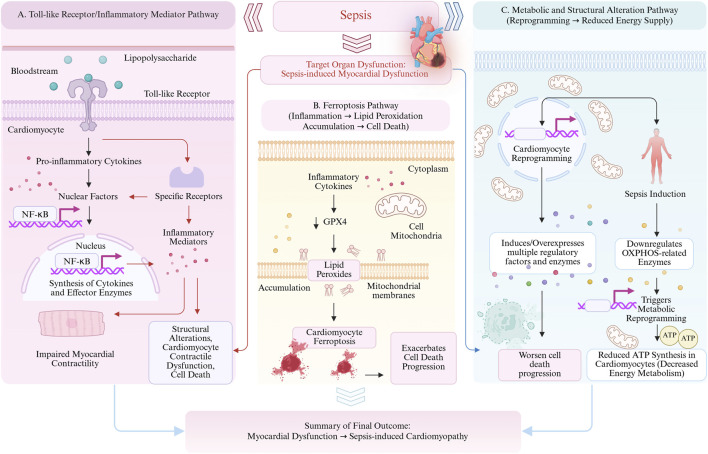
Sepsis-induced cardiomyopathy involves ferroptosis, metabolic reprogramming, and mitochondrial dysfunction leading to contractile failure. Downregulation of GPX4 by inflammatory cytokines induces lipid peroxidation, triggering cardiomyocyte ferroptosis prior to apoptosis or necrosis. LPS-TLR and cytokine signaling promote NF-κB nuclear translocation, upregulating pro-death enzymes while suppressing oxidative phosphorylation enzymes. This leads to a sudden ATP depletion, contractile protein damage, and mitochondrial dysfunction, with metabolic reprogramming synergistically exacerbating myocardial contractile failure.

Inflammatory cytokines induce the accumulation of lipid peroxidation by downregulating GPX4 expression, a pathological change that may occur prior to traditional apoptosis and necrosis (Huang Y. et al., 2022). During the onset of SIMD, cardiomyocyte reprogramming mediated by the induction or overexpression of various regulatory factors and enzymes plays a pivotal role in accelerating the process of cell death. This mechanism is primarily characterized by LPS binding to Toll-like receptors, alongside the interaction between pro-inflammatory cytokines and their specific receptors. Collectively, the aforementioned processes promote nuclear translocation of transcription factors, activate synthesis of cytokines and various effector enzymes, and ultimately lead to impaired myocardial contractile function. Meanwhile, downregulation of enzymes associated with oxidative phosphorylation induces metabolic reprogramming, resulting in reduced intracellular ATP production in cardiomyocytes (Hobai, 2023) (Figure 5, three major pathogenic mechanisms of SIMD).

Pyroptosis mediated by GSDMD plays a fundamental role in myocardial injury associated with sepsis. Novel GSDMD inhibitor GI-Y1 and its macrophage membrane-coated nanoparticles (GI-Y1@MM-NPs) have emerged as promising therapeutic strategies. Experiments showed that GI-Y1 could selectively inhibit GSDMD activity, significantly improve cardiac function and reduce myocardial injury in LPS and CLP-induced sepsis mice, and this protective effect completely disappeared in the GSDMD knockout model, confirming that its effect depended on the GSDMD pathway. Further optimized GI-Y1@MM-NPs showed stronger anti-inflammatory and cardioprotective effects, suggesting that targeting GSDMD-mediated pyroptosis inhibition may be a new avenue for treating sepsis-related myocardial dysfunction (Shi et al., 2025). It is worth noting that GSDMD-mediated pyroptosis exacerbates the inflammatory response by releasing pro-inflammatory factors such as IL-1β, and necrosulfonamide (NSA) can directly bind to and inhibit the pore formation activity of GSDMD, effectively block the pyroptosis process, and confirm its therapeutic potential in sepsis models, suggesting that GSDMD inhibitors may become a novel therapeutic strategy for inflammatory diseases (Rathkey et al., 2018).

As a systemic inflammatory response syndrome, sepsis often causes multiple MODS in the heart, lung, kidney, liver, and its pathophysiological mechanisms are complex and diverse, involving uncontrolled inflammatory responses, microcirculatory perfusion disorders, endothelial cell damage, mitochondrial dysfunction and cell death pathway activation, especially with the participation of PCD pathways such as pyroptosis and necrotizing apoptosis, which further exacerbates tissue damage. It is worth noting that there is significant heterogeneity in the failure patterns of different target organs: cardiac dysfunction is mostly manifested by decreased contractility, lung injury is mainly characterized by ARDS, and liver and kidney failure is more prone to metabolic disorders and accumulation of toxic substances. This difference is not only related to the fragility and degree of damage of specific organs, but also to the rate of progression, compensatory ability and repair potential of the disease. Therefore, future research needs to integrate multi-omics techniques and cross-organ comparative analysis to deeply explore the spatiotemporal specific molecular mechanisms of sepsis-related organ dysfunction, and combine organoid models and large-scale clinical cohort validation to establish organ-specific precision intervention strategies, so as to improve the overall prognosis of sepsis patients.

## 5 Therapeutic strategies targeting cell death

### 5.1 Pharmacological research targeting apoptosis

When sepsis occurs, apoptosis, a caspase-dependent type of PCD, is commonly blamed for the failure of critical organs like the kidneys, liver, and lungs (Li and Yuan, 2008; Fulda and Debatin, 2006; D'Arcy, 2019). Researchers have determined that cytokines are effective pharmacological treatments for this pathological process. For instance, by suppressing pro-apoptotic factors and upregulating the anti-apoptotic marker Bcl-2, IL-7 has a protective effect. Preventing the depletion of T-cells that usually follows excessive apoptosis requires this molecular change (Unsinger et al., 2010; Li et al., 2010).

Bcl-2 family modulators and caspase inhibitors have been the focus of primary research on the therapeutic potential of apoptotic pathways (Li Y. et al., 2023). In particular, the severity of LPS-induced endotoxemia has been shown to be reduced by the administration of the caspase inhibitor Z-VAD-FMK. Myeloid-derived suppressor cells can then mediate the downregulation of macrophage-driven inflammatory responses by inducing macrophage necroptosis (Li X. et al., 2019). Furthermore, the Bcl-2 targeting drug fisetin effectively reduces the number of TUNEL-positive apoptotic cells in the kidneys of mice with LPS-induced SA-AKI. Research indicates that Fisetin provides a dual benefit by countering both inflammation and apoptosis within renal tissues. This is achieved primarily through the inhibition of Bcl-2, BAX, and cleaved caspase-3, which are critical mediators of the apoptotic pathway (Ren et al., 2020).

### 5.2 Clinical applications of autophagy modulators

Autophagy in sepsis exhibits a dual nature: moderate autophagy can remove denatured, damaged, and non-functional proteins as well as subcellular organelles, thereby maintaining cellular homeostasis; however, excessive autophagy may lead to cellular autodigestion and exacerbate tissue damage (Matsuzawa-Ishimoto et al., 2018; Gao et al., 2019). Studies have shown that in the early stages of sepsis, autophagy may be enhanced through mTOR-dependent or independent pathways; however, in the later stages, there is an impairment in the clearance of autophagosomes (Hsu and Wang, 2020).

The mTOR inhibitor rapamycin has been shown to enhance autophagy and reduce myocardial dysfunction in animal models of sepsis (Fang et al., 2024), while chloroquine exhibits protective effects in conditions of excessive autophagy (Fang et al., 2013). Nevertheless, precisely regulating autophagy at different stages of the disease remains a significant challenge. Current clinical trials primarily focus on investigating the potential protective effects of mTOR inhibitors in sepsis; however, their safety and long-term efficacy still require further evaluation.

### 5.3 Prospects of immunomodulatory therapies

Sepsis is characterized by early hyperinflammation and late immunosuppression, and pyroptosis and necrotizing apoptosis play a key role in this process (Hotchkiss et al., 2009; Jiang et al., 2018). In recent years, immunomodulatory strategies targeting cell death-related inflammatory pathways have gradually attracted attention. The IL-1β antagonist anakinra has shown an improvement in sepsis-associated macrophage activation syndrome in some clinical trials, significantly improving patient survival (Shakoory et al., 2016). In addition, PD-1/PD-L1 immune checkpoint inhibitors, such as nivolumab, have shown some effect in reversing T cell depletion and improving survival in advanced sepsis, but caution should be exercised against the potential risk of over-immune activation (Cao et al., 2023; Chen et al., 2023; Wang et al., 2021). In the future, individualized immunomodulation protocols combined with biomarkers may become a new direction for sepsis treatment.

The timing of cell death-targeted therapies in sepsis requires precise alignment with the disease’s dynamic biphasic nature, characterized by an early hyperinflammatory phase followed by a late immunosuppressive phase. During the initial hours to days following sepsis onset, the hyperinflammatory “cytokine storm” dominates, driven by pyroptosis, necroptosis, and ferroptosis that amplify tissue damage and organ dysfunction. This narrow window represents the critical period for administering inhibitors targeting these pro-inflammatory cell death pathways, such as NLRP3 inflammasome inhibitors (MCC950, CY-09), necroptosis inhibitors (Necrostatin-1, necrosulfonamide), and ferroptosis inhibitors (ferrostatin-1, iron chelators), to prevent the establishment of the necro-inflammatory amplification loop (Linkermann et al., 2014). However, immunostimulatory therapies must be approached cautiously during this phase to avoid exacerbating inflammatory injury (Ono et al., 2018).

As sepsis progresses beyond 48–72 h, the pathophysiological landscape shifts dramatically toward immunosuppression, marked by extensive lymphocyte apoptosis, T cell exhaustion, and myeloid cell dysfunction. This late phase, which can persist for days to weeks, demands a fundamentally different therapeutic strategy focused on preventing or reversing immunoparalysis. During this period, apoptosis inhibitors (such as those targeting caspase-3 or the Bcl-2 family) and immune checkpoint inhibitors (anti-PD-1/PD-L1) become the priority to preserve adaptive immune cell populations and restore host defense capabilities (Islam et al., 2024). Notably, clinical evidence suggests that the immunosuppressive phase provides a broader therapeutic window for intervention, as patients who survive the initial inflammatory surge often succumb to secondary infections resulting from immune dysfunction (Ono et al., 2018).

**FIGURE 6:**
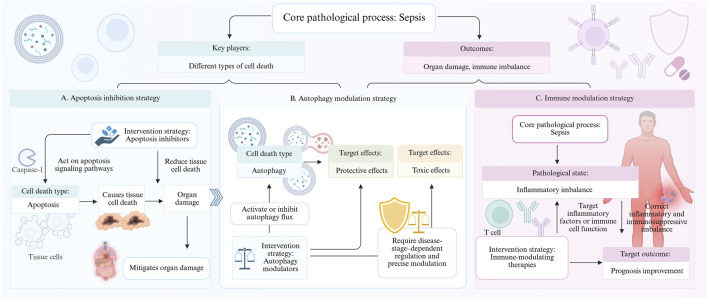
Targeted modulation of cell death pathways and immune balance holds therapeutic potential for sepsis. Sepsis is characterized by a cellular death storm, where apoptosis, autophagy, and immune dysregulation intertwine to form a network of organ damage. Apoptosis inhibitors block caspases to reduce cell loss, autophagy modulators fine-tune the protection-toxicity balance depending on disease phase, and immunotherapy maintains the equilibrium between pro- and anti-inflammatory responses synergistically mitigating multiorgan failure. Future strategies should leverage biomarkers to guide personalized, organ-targeted combination therapies, breaking free from single-pathway limitations and advancing toward a new paradigm of multi-mechanism synergistic intervention.

Different types of cell death in sepsis play a key role in organ damage and immune imbalance, and intervention strategies targeting these pathways show promising therapeutic prospects. Aptoposis inhibitors alleviate organ damage by reducing tissue cell death; Autophagy modulators need to be precisely regulated according to the disease stage to balance their protective and toxic effects; Immunomodulatory therapies improve prognosis by correcting the imbalance between inflammation and immunosuppression. However, existing therapies still face challenges such as insufficient specificity, timing dependence, and potential side effects. Future research should combine multimodal biomarkers and dynamic monitoring techniques to develop individualized, organ-targeted combination therapy to achieve the leap from single-pathway inhibition to multi-mechanism synergistic intervention in sepsis treatment (Figure 6, treatment strategies for sepsis).

## 6 Biomarkers of organ injury

### 6.1 Research progress in cell death-related biomarkers

Regarding the selection criteria for organ-specific biomarkers in sepsis, an ideal biomarker should exhibit high sensitivity and specificity, be easily detectable, and provide real-time information on the patient’s clinical status (Shin, 2025). At the organ-specific level, selection criteria should comprehensively consider tissue-specific expression, early predictive capability, pathophysiological relevance to organ dysfunction, and value for dynamic monitoring. For example, circulating miR-122, a tissue-specific RNA, is expressed at approximately 70% in the liver and at very low levels in other tissues, enabling earlier and more specific prediction of acute liver injury compared to conventional transaminases (AST/ALT) (Rahmel et al., 2018). Furthermore, biomarkers should reflect complementary mechanisms of organ injury; for instance, adrenomedullin reflects cardiovascular and endothelial dysfunction, proenkephalin A correlates with glomerular filtration rate, and DPP3 indicates cellular death. The combined application of these markers facilitates the development of a more precise system for assessing organ function (Bolanaki et al., 2024).

**FIGURE 7:**
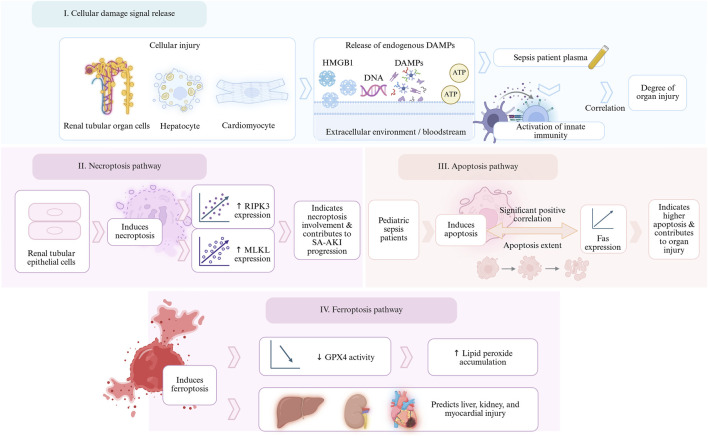
Molecular mechanism of sepsis-induced organ injury. Damaged cells release HMGB1, DNA, ATP, and other DAMPs into the bloodstream, with concentrations correlating with organ injury. These molecules first activate innate immunity, amplifying inflammation. In renal tubules, RIPK3-MLKL upregulation triggers necroptosis, while in pediatric cases, Fas overexpression drives apoptosis. In the liver, heart, and kidneys, GPX4 levels plummet, leading to lipid peroxidation accumulation and triggering ferroptosis. The overlap of these three programmed cell death pathways (necroptosis, apoptosis, and ferroptosis) provides molecular targets for precision intervention, such as RIPK3, Fas, and GPX4.

Cell damage can release endogenous DAMPs that activate innate immunity, and these molecules are significantly elevated in the plasma of sepsis patients and are associated with the degree of organ damage (Zhang et al., 2010). Necrotizing apoptosis-related markers such as RIPK3 and MLKL are increased in SA-AKI patients, indicating that necrotizing apoptosis of TECs is involved in the development of SA-AKI (Wu et al., 2022). In addition, in children with sepsis, the degree of apoptosis was significantly positively correlated with the expression of apoptosis-related molecules Fas (Qinfen et al., 2025). Decreased activity of GPX4, a characteristic marker of ferroptosis, and lipid peroxide accumulation may indicate the occurrence of liver, kidney, and myocardial injury (Yang et al., 2016; Hemmati et al., 2025; Wang S. et al., 2024). The study of these markers provides insight into the molecular mechanisms of organ damage in sepsis and may be targets for therapeutic interventions (Figure 7, mechanisms of cell death in organ damage in sepsis).

### 6.2 Clinical applications of biomarkers in diagnosis

At present, some biomarkers have shown practical value in the clinical evaluation of sepsis. As an advanced inflammatory mediator, the plasma levels of HMGB1 are not only associated with sepsis severity but also with predictive risk of MODS (Wang et al., 2014). Neutrophil gelatinase-associated lipocalin (NGAL) and liver-type fatty acid-binding protein (L-FABP) have been studied as early markers of acute kidney injury, and their elevation predates traditional indicators such as serum creatinine, contributing to clinician intervention earlier (Tang et al., 2017).

In addition, procalcitonin (PCT) and C-reactive protein (CRP), as routine indicators (Beqja-Lika et al., 2013), for sepsis diagnosis, can improve the accuracy of predicting the risk of organ damage in patients when combined with novel cell death markers. However, the sensitivity and specificity of a single biomarker are still limited, and multi-marker joint analysis needs to be explored to improve diagnostic efficiency in the future.

However, there are significant technical barriers to monitoring the dynamic process of cell death in real time. While flow cytometry can detect the expression of lymphocyte subsets and immune checkpoint molecules such as PD-1, it requires specialized laboratory equipment and experienced technicians, and sample handling can lead to cell loss and attenuation of fluorescent markers, affecting quantitative accuracy (Liu et al., 2017). More importantly, most of the existing detection methods are measured at a single time point, and it is difficult to capture the biphasic dynamic characteristics of sepsis cell death - that is, the early stage of high inflammation and the late stage of immunosuppression. Although immune status markers such as lymphocyte count and monocyte HLA-DR can reflect the degree of immunosuppression, their detection lacks standardization and cannot distinguish the specific contributions of different cell death modes (Misra et al., 2020).

### 6.3 Potential value of emerging biomarkers

In recent years, many emerging biomarkers have been discovered, which may bring breakthroughs in the precise diagnosis and treatment of sepsis organ damage. For example, heat shock proteins (HSPs) carried by extracellular vesicles (EVs) reduce cell bypass permeability of primary human brain endothelial cells under hypoxic glucose deprivation conditions and predict the progression of brain injury (Dave et al., 2023). Citrullinated histone H3 (CitH3), a component related to NETs, is not only involved in thrombosis, but is also closely related to tissue damage and chronic inflammation, and is considered one of the most reliable and specific biomarkers for NETs formation, which can indirectly assess the intensity of inflammatory and immune processes triggered by neutrophil activation by detecting its serum concentration (Ouyang et al., 2025). In addition, single-cell RNA sequencing-based studies have found that by identifying unique cell subsets and cell death-related molecules associated with sepsis, it can provide potential targets for individualized therapy (Shen et al., 2025). Although the research on these novel markers is still in its early stages, their potential in predicting organ damage, guiding immunomodulatory therapy, and evaluating treatment efficacy deserves further exploration.

The study of biomarkers related to cell death provides an important basis for early diagnosis, risk stratification and precise intervention of sepsis organ damage. Existing markers have been partially applied in clinical practice, but still have limitations in terms of sensitivity or lack of specificity. Emerging technologies are driving the development of more specific markers that are expected to play a key role in the prediction and therapeutic monitoring of organ damage in sepsis. Future research should focus on multi-omics integration and dynamic monitoring, and establish a biomarker combination based on cell death mechanisms to improve the early warning ability and personalized treatment guidance of sepsis organ damage, thereby improving patient prognosis.

## 7 Discussion

The complexity of sepsis is not only reflected in the dynamic changes of its pathophysiological processes, but also in the networked regulation of different forms of cell death mechanisms in MODS. PCD pathways such as apoptosis, autophagy, necrotizing apoptosis, ferroptosis, and pyroptosis intertwine in sepsis, forming a vicious circle that ultimately leads to immune imbalance and organ failure. Notably, excessive inhibition of pro-inflammatory death may impair pathogen clearance, while simply blocking immune apoptosis may delay the resolution of inflammation. Therefore, ideal treatment strategies should be both spatiotemporal specific, such as inhibiting pyroptosis in the early stages of sepsis to mitigate the inflammatory storm and blocking lymphocyte apoptosis to rebuild immune function in the later stages.

GSDMD-mediated membrane perforation can not only trigger typical pyroptosis, but also activate the cGAS-STING pathway by releasing mtDNA, thereby promoting NLRP3 inflammasome activation, forming a positive feedback loop of “pyroptosis-STING-inflammasomes”, and exacerbating tissue damage. In addition, lipid peroxidation caused by ferroptosis not only disrupts the antioxidant defense system, but may also indirectly aggravate necrotizing apoptosis and inflammatory responses. These findings not only explain why specific organs tend to die in certain patterns, but also provide a theoretical basis for the development of multi-target combination therapies. However, balancing inflammation control with cellular protection at different stages of the disease remains a key challenge, with premature inhibition of pro-inflammatory death potentially weakening pathogen clearance and late intervention making it difficult to reverse organ damage. In addition, the regulation of death pathways in animal models is often highly simplified and cannot fully mimic the heterogeneity of human sepsis, especially the effects of comorbidities or genetic background. For example, the apoptosis-necrotosis-apoptotic transition induced by caspase-8 deletion in mouse models may be very different in humans due to epigenetic modifications or microbiome interference. In addition, it is difficult to reproduce common opportunistic or mixed infection scenarios in clinical practice. This network-based regulation suggests that intervention for a single pathway of death may have limited effects, and future treatment strategies need to consider multi-target combined regulation.

Although important progress has been made in the role of cell death mechanisms in sepsis, several key issues remain to be addressed. First, the preference of different organs for cell death patterns is unclear, such as why lung injury is more likely to be accompanied by pyroptosis, while liver and kidney injury is more closely related to ferroptosis. Second, most of the existing research is based on animal models, but the heterogeneity and complexity of human sepsis may limit its clinical translation. Therefore, it is crucial to utilize tissue samples from organoids or patient sources for validation. In addition, drugs targeting the cell death pathway (necrotosis inhibitor Nec-1, ferroptosis inhibitor Fer-1) have shown potential in preclinical studies, but their safety and long-term efficacy still need to be further evaluated (Cao and Mu, 2021; Zhang L. et al., 2024). Finally, how to integrate these basic research results into the precise classification and individualized treatment of sepsis is the core challenge of future research.

Pathogen burden and infection site further regulate the selection of cell death modes: a high bacterial load tends to induce neutrophil NETosis and macrophage pyroptosis to rapidly clear pathogens, whereas persistent infections lead to T cell exhaustion and immunosuppressive apoptosis. Different pathogens also exhibit organ tropism; for example, *Pseudomonas aeruginosa* preferentially induces necroptosis in alveolar epithelial cells, while *Candida* albicans more readily triggers PANoptosis in the kidneys. This organ specificity is closely associated with the local immune microenvironment and the distribution of pathogen receptors. Understanding these pathogen–cell death interaction mechanisms provides a theoretical foundation for developing precision intervention strategies targeting specific pathogens.

The prevention and treatment of sepsis involves a concerted effort in various fields such as critical care medicine, microbiology, immunology, and pharmacology. For example, immunomodulatory therapies need to be combined with infection control to avoid over-suppressing the immune response leading to pathogen clearance disorders (Liu D. et al., 2022), while targeted therapies for cell death need to be combined with traditional methods such as hemodynamic support and anticoagulation (Bao et al., 2025). The study showed that AI-assisted early warning systems for sepsis and dynamic monitoring of biomarkers provided new tools for multidisciplinary collaboration (Aityan et al., 2025; Papareddy et al., 2025). These systems provide new tools for multidisciplinary collaboration and can help improve clinical outcomes in patients with sepsis.

Future research urgently needs to make breakthroughs in three directions. Firstly, the organoid or *ex vivo* organ perfusion system was used to simulate the preference for death response in human tissues, and the division of labor of different cell subsets in the death network was analyzed by single-cell sequencing technology. Second, optimize the design of existing inhibitors, such as organ-specific targeting through nanodelivery systems. Finally, the dynamic monitoring data of clinical biomarkers and the artificial intelligence prediction model were integrated to distinguish the dominant subtypes of the death pathway of patients, so as to formulate individualized intervention strategies.

In conclusion, the paradigm of treatment is radically changing from basic supportive care to targeted molecular regulation as our knowledge of the mechanisms underlying cell death in sepsis grows. Precision medicine frameworks that target the cell death network have the potential to revolutionise the management of sepsis, despite the lengthy path to widespread clinical application. These tactics rely on a multimodal strategy that incorporates superior model systems, multi-omics integration, and a strong multidisciplinary cooperative effort. Additionally, the developments in this area will provide a fundamental framework for studying other inflammatory or infectious diseases.

## Glossary

AECs: alveolar epithelial cells
ALDH2: aldehyde dehydrogenase 2
ARDS: acute respiratory distress syndrome
Arp2/3: actin-related protein 2/3
CitH3: Citrullinated histone H3
CLP: cecal ligation and puncture
CRP: C-reactive protein
CXCR5: C-X-C motif chemokine receptor 5
CYLD: cylindromatosis
DAMPs: damage-associated molecular patterns
ER: endoplasmic reticulum
EVs: extracellular vesicles
FH: fumarate hydratase
GPX4: glutathione peroxidase 4
GSDMD: gasdermin D
HK2: hexokinase 2
HSC: hepatic stellate cell
HSPs: heat shock proteins
IFN-I: type I interferons
iNOs: inducible nitric oxide synthase
LCN2: lipocalin-2
L-FABP: liver-type fatty acid-binding protein
lncRNAs: long non-coding RNAs
LPS: lipopolysaccharide
MIF: macrophage migration inhibitory factor
miRNAs: microRNAs
MLKL: mixed lineage kinase domain-like protein
**MODS**: multiple organ dysfunction syndrome
mtDNA: mitochondrial DNA
mTOR: mammalian target of rapamycin
NETs: neutrophil extracellular traps
NGAL: neutrophil gelatinase-associated lipocalin
Nrf2: nuclear factor erythroid 2-related factor 2
NSA: necrosulfonamide
PAMPs: pathogen-associated molecular patterns
PCD: programmed cell death
PCT: procalcitonin
PD-1: programmed cell death protein 1
PMN: polymorphonuclear neutrophils
RAGE: receptor for advanced glycation end-products
RIPK3: receptor interacting protein kinase 3
ROS: reactive oxygen species
SA-AKI: sepsis-associated acute kidney injury
SA-ALI: sepsis-associated acute lung injury
SAE: sepsis-associated encephalopathy
SALI: sepsis-associated liver injury
SIMD: sepsis-induced myocardial dysfunction
STING: stimulator of interferon genes
TECs: tubular epithelial cells
TNF: tumor necrosis factor
TRAIL: tumor necrosis factor-related apoptosis-inducing ligand.
